# The Separatrix Algorithm for Synthesis and Analysis of Stochastic Simulations with Applications in Disease Modeling

**DOI:** 10.1371/journal.pone.0103467

**Published:** 2014-07-31

**Authors:** Daniel J. Klein, Michael Baym, Philip Eckhoff

**Affiliations:** 1 Institute for Disease Modeling, Bellevue, Washington, United States of America; 2 Department of Systems Biology, Harvard Medical School, Boston, Massachusetts, United States of America; 3 Department of Mathematics, Massachusetts Institute of Technology, Boston, Massachusetts, United States of America; University College London, United Kingdom

## Abstract

Decision makers in epidemiology and other disciplines are faced with the daunting challenge of designing interventions that will be successful with high probability and robust against a multitude of uncertainties. To facilitate the decision making process in the context of a goal-oriented objective (e.g., eradicate polio by 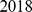), stochastic models can be used to map the probability of achieving the goal as a function of parameters. Each run of a stochastic model can be viewed as a Bernoulli trial in which “success” is returned if and only if the goal is achieved in simulation. However, each run can take a significant amount of time to complete, and many replicates are required to characterize each point in parameter space, so specialized algorithms are required to locate desirable interventions. To address this need, we present the Separatrix Algorithm, which strategically locates parameter combinations that are expected to achieve the goal with a user-specified probability of success (e.g. 95%). Technically, the algorithm iteratively combines density-corrected binary kernel regression with a novel information-gathering experiment design to produce results that are asymptotically correct and work well in practice. The Separatrix Algorithm is demonstrated on several test problems, and on a detailed individual-based simulation of malaria.

## Introduction

Decision makers are tasked with the challenging job of determining how best to achieve specific program goals despite large amounts of uncertainty. In public health, for example, policy makers must decide which interventions to use and when, as well as the demographic sub-population to target, so as to achieve programmatic goals. Global eradication of a disease is a particularly relevant example of one such goal. Malaria is the target of a Global Eradication Campaign announced in 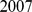, and a successful campaign will most likely require combining several different interventions, with the particular combination tailored to the local transmission setting [1]. Similarly, poliomyelitis has been the target of a global eradication initiative since 1988 [2], wherein oral [3] and inactivated [4] polio vaccines are distributed through various routine and supplementary immunization activities [5].

To achieve these goals with high probability, complex interactions between decisions (e.g. deploy bednets to 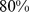 of the population) and mechanistic uncertainties (e.g. the extent to which mosquitoes feed indoors) must be considered systematically. Computer models enable these interactions to be simulated thousands of times *in silico* before trying in the real world. When using a model to evaluate an objective, each stochastic run can be viewed as a Bernoulli trial in which “success” is returned if and only if the programmatic goals are achieved in simulation. Many trials (simulations) are required to adequately characterize the underlying probability of success for each parameter configuration. Mapping the success probability across parameter space is highly desired, however the operational regime consists of a stochastic model that can take hours to produce each binary outcome. Efficient algorithms are thus required to best use limited computational resources, particularly now that increasingly-detailed computational models are being used directly in guiding policy decisions in HIV [6], [ 7], and in campaign design for influenza [8]–[11], polio [12], and malaria [13]–[17].

Fortunately, many campaigns do not require achieving full population coverage in order to succeed in interrupting transmission, with the required level depending on the disease's basic reproductive number. Combined with the phenomenon that increasing coverage becomes increasingly expensive for marginal benefits, threshold phenomenon suggest that the probability success can saturate close to 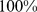 while campaign costs continue to rise with coverage. Thus, regions of campaign and parameter space in which success is almost guaranteed are not particularly interesting. Similarly, regions in which the probability of success is low are uninteresting. The key algorithmic challenge lies in concentrating computational resources in regions of parameter space in which the success probability is near an intermediate target value, such as 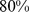 or 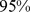.

Related work has primarily focused on analytical techniques for deterministic simulation models. For simulations of this type, the only source of output variation comes from (epistemic) uncertainty about parameter values. Epistemic variation is classically analyzed for uncertainty and sensitivity using numerical sampling methods such as Latin Hypercube Sampling (LHS), see [18], [19]. Stochastic models, in which aleatory uncertainty stemming from randomized components is included, are more difficult to analyze because each parameter configuration maps to a multitude of outcomes. Nonetheless, some of the techniques from deterministic model analysis have been adapted to work with stochastic simulation models by running the model many times for each parameter configuration, and using the average as if it were deterministic [20]–[25]. A second approach to the analysis of stochastic models is the response surface methodology, see [26]–[30]. The main idea is to fit an easy-to-evaluate metamodel [31] to the mean of a collection of simulation runs for analysis [32] or optimization [33]. While typically based on polynomials, numerous metamodels have been explored including radial basis functions [34], neural networks [35], thin-plate splines [36], support vector machines, and Gaussian processes (kriging) [37], [38].

Another area of related work to consider is experiment design, the problem of choosing which simulations to run on the next iteration. Simple designs include factorial and fractional factorial designs [39] that improve upon varying one parameter at a time by exploring locally extreme variations. Distance maximizing and information theoretic approaches have been explored [40], with much of the work directed towards designing computer experiments [41]–[44]. More advanced and computationally intensive approaches to experiment design include a class of algorithms that seek to optimize the expected information returned by the samples by minimizing a loss function [45], [46] or maximizing the expected information gain [47]–[49].

None of the above work satisfactorily addresses the needs of decision makers, who need efficient algorithms to draw meaningful conclusions from detailed stochastic simulations with respect to a goal-oriented objective. To address these needs, we present the Separatrix Algorithm, which directs computational resources towards identifying and resolving combinations of policies and uncertain model parameters that will achieve the goal successfully with a given probability. The algorithm was named after a mathematical term, separatrix, representing a boundary separating behavioral modes. Here, we use the term as a synonym for the desired isocline of the underlying success probability function. While in this work we present the Separatrix Algorithm in the context of computational epidemiology, the methods extend directly to other computational disciplines and further to the scientific domain, wherein laboratory experimentation replaces computer simulation.

**Figure 1:**
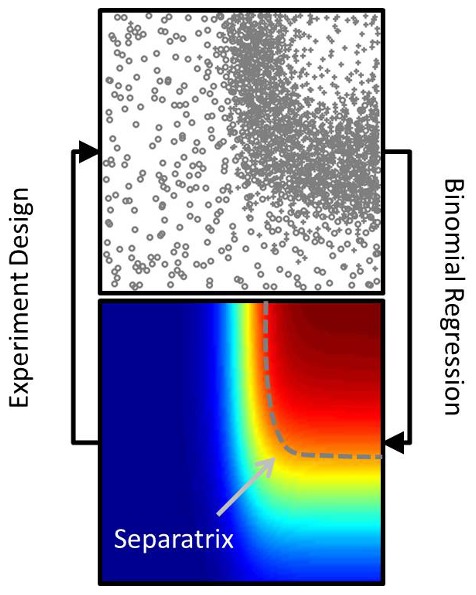
The Separatrix Algorithm addresses two main sub-problems. The first is to use observed binary outcomes (top) to estimate the probability of success (bottom), and the second is to choose new points to sample. This is done so as to identify a particular isocline, called the separatrix, as illustrated by the dashed gray line.

The main contributions of this work include (1) an iterative approach to stochastic model evaluation in the context of a goal-oriented objective that consists primarily of (2) a consistent kernel-based regression algorithm that estimates the full distribution of the probability of achieving the goal at a point based on observed simulation outcomes, and (3) a design-of-experiments algorithm that places the next 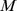 sample points so as to strategically gain information about the separatrix. These elements are illustrated in Figure 1.

## Methods

The Separatrix Algorithm combines binomial regression, based on novel kernel methods, with an experiment design procedure, called igBDOE, that maximizes expected information gain. The algorithm progresses by iteratively choosing new parameter configurations to simulate (i.e. sample points) from a 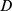-dimensional box, called the *parameter space* or *sample space*, that we will denote by 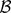.

Simulations that are complete will be denoted by 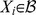 and 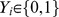, 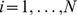, representing the sample points and outcomes, where 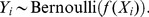
(1)


The success probability function, denoted by 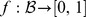, represents the true probability of achieving the goal on a particular simulation trial. While this function is unknown in practice (the purpose of the Separatrix Algorithm is to estimate this function near the user-specified isocline), it is safe to assume that 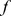 is sufficiently regular in terms of continuity and differentiability.

Let 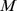 denote the number of sample points to be selected on each iteration, and note that 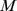 should be selected to maximally leverage parallel computing resources. These “next points” to sample will be selected by the Separatrix Algorithm so as to gather information about an isocline, denoted by 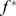, of the underlying success probability function. We will refer to this isocline of the success probability function as the separatrix, see Figure 1.

The first samples are selected using a space filling algorithm, such as LHS. Then, the main iteration loop consists of two primary steps. The *inference step* takes available samples, 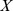, and outcomes, 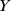, as inputs, and returns a probability density function for the probability of success at one or more *inference points*. The *design of experiments step* uses the data to select subsequent sample points. Each of these algorithms will be described in detail in the following sections, and are summarized in Algorithm Summary S1.

### Stochastic Inference using Kernel Regression

The inference portion of the Separatrix Algorithm uses a novel variation of kernel regression to estimate the *distribution* of the probability of success at a specified inference point, 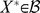. This differs from most kernel methods, which simply estimate a single value, representative of the mean, at each inference point.

The output from each simulation is passed through a Boolean test function that returns “true” only if the user-specified goal is achieved. Because each simulation is an independent Bernoulli trial, if one or more simulation are run with a *fixed* parameter configuration, the resulting probability of success after observing 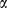 successes and 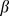 failures would have a beta distribution, 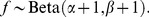
(2)


However, it is inefficient to run multiple simulations with a fixed parameter configuration, and we often wish to compute the distribution at a point for which no outcomes have been observed.

Our approach to the inference problem at 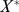 is to first estimate the effective number of observed successes, 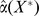, and failures, 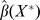, using kernel methods, and then plug these estimates into the beta distribution, 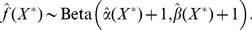
(3)


Kernel methods, commonly used in density estimation and regression, compute the result value as a (kernel) weighted average of “nearby” values. Nearness is determined by the spatial scale of the kernel, called the bandwidth. Many techniques have been proposed for selecting the kernel bandwidth, here we derive the kernel scale from the distance to the 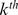 nearest neighbor (KNN).

When the values are sampled uniformly, classical kernel methods are consistent, meaning that the estimate is asymptotically correct in probability. However, direct application of these concepts results in an unacceptably large bias when the data are not sampled uniformly. This bias in classical kernel methods is unacceptable for direct application in the Separatrix problem because the very objective of the algorithm is to concentrate samples near the separatrix, resulting in a non-uniform sampling density that becomes increasingly non-uniform as additional samples are collected.

To minimize bias with highly non-uniform sampling, the inference portion of the Separatrix Algorithm uses a density correction factor in the kernel. Bias correction has been used in binary kernel regression by Hazelton [50], however our approach differs. We proceed by describing the two main sub-steps of the inference portion of the Separatrix Algorithm: (1) estimate the sample point density using standard kernel methods, and (2) apply a density-corrected kernel to estimate the number of successes and failures, which then feeds into a beta distribution to finally approximate the distribution of the probability of success at each inference point.

#### Density Estimation

The density estimator uses standard variable kernel smoothing techniques [51] to produce an estimate of the sample point density at each sample point. While any consistent density estimator could be used, variable kernel methods are known to produce a bona fide density estimate that is globally consistent, however the edges converge at a slower rate [52].

The density estimate at point 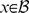 is computed as 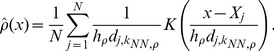
(4)


Here, 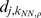 is the distance from sample point 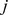 to the 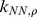-th nearest sample point. The variable kernel method employs a bandwidth that is proportional, with constant 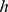, to this distance. Many authors have proposed methods to select 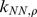 automatically; we use the parametric form 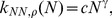
(5)for constants 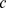 and 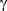.

Due to extreme density variations driven by the desire to sample near the separatrix, we replace the global 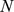 in (5) with a local estimate of the number of sample points at each inference point, 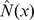, using a scale model, 
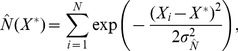
(6)in which 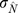 is a fixed bandwidth. Note the lack of kernel normalization above. Instead, the kernel is one when 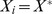 so that a sample at 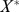 counts as one “effective” sample, but samples further from 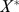 count only partially in the total.

#### Success Probability Distribution Inference

With the density estimates available, the effective number of successes and failures at 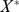 are calculated as, 
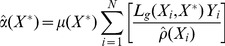
(7)

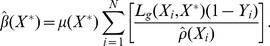
(8)


Here, 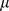 is a scaling factor (to be explained shortly), and 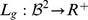 is a kernel function with bandwidth 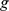. We use a squared exponential kernel with a simple S-type (i.e. diagonal) bandwidth matrix, for which the scaling is determined by the distance to the 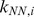-th nearest neighbor, as in (5), although the parameters are not necessarily the same. Again, we replace the global 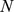 by 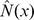 from the scale model.

The scaling constant, denoted by 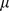, is selected so that the number of success and failure outcomes at 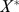 sums to the estimated “effective number of samples” at 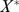 from the scale model (6), 
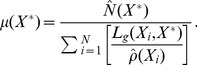
(9)


This scaling by 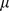 in the calculation of 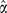 and 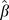 has no impact on mode of the beta distribution (8), but does affect higher order moments.

The distribution of the probability of achieving the goal at each test point is approximated by a beta distribution (3). The approximation comes from the fact that the true posterior has a beta distribution only for repeated simulations (i.e. fixed parameters).

### Design of Subsequent Experiments

The second main step of the Separatrix Algorithm is a design-of-experiments (DOE) procedure that selects 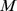 new sample points in a manner that actively focuses computational effort on interesting regions of parameter space. This is counter to traditional factorial, space-filling, and variance-reducing approaches, which sample globally. Traditional Bayesian design-of-experiments (BDOE) methodology, see the original work [53] or a review [54], also places samples globally. However, samples in BDOE are actively placed so as to maximize the expected information gain, as measured by a Kullback-Liebler (KL) pseudo-metric. The experiment design portion of the Separatrix Algorithm uses an “interest guided” (igBDOE) approach in which the distributions used in the KL calculation are tailored in a novel way that encourages subsequent samples to focus on identifying the separatrix.

More specifically, the BDOE methodology chooses new input configurations, 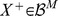, that are expected to maximally distinguish the posterior distribution, which includes the 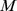 proposed samples, from the prior distribution, which does not: 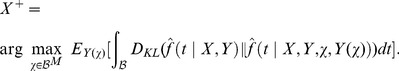
(10)


Here, 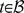 is a test point at which the prior and posterior distributions are to be compared, 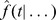 is the inferred success probability distribution at 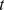, 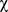 is the collection of 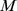 sample points in consideration, and 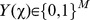 is the corresponding outcome vector. In a small abuse of notation, 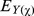 denotes expectation with respect to the *distribution* over outcomes at 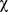, which in turn is calculated from the expected value of 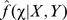. Finally, 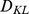 denotes the Kullback-Liebler divergence between distributions 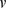 and 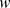, 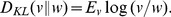
(11)


Without modification, traditional Bayesian experiment design selects new inputs that are globally important.

In contrast, the *interest-guided BDOE* (igBDOE) algorithm used in the Separatrix Algorithm focuses subsequent samples on the regions of parameter space for which the success probability is near 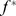. Rather than applying the traditional BDOE methodology to the beta distribution from the inference algorithm, igBDOE applies a similar approach to a discretization we call the *interest distribution*, 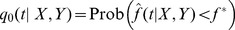
(12)

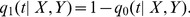
(13)


Here 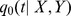 is the total probability mass of distribution 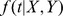 that lies below the interest level, and 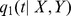 is the mass above the interest level. Similarly, 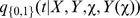 is the total probability mass of distribution 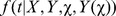 that lies below (above) the interest level. In this way, the experiment design procedure selects new inputs that are expected to move mass across separatrix. As a consequence, the procedure samples primarily in and around the desired isocline, as these locations have the greatest potential to change the interest distribution, and occasionally samples far away when uncertainty is sufficiently high.

Practically, Bayesian approaches to experiment design tend to be computationally intensive. To make the computation tractable, we apply a number of simplifications. In place of the integral in (10), we restrict consideration to 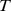 test points 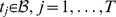. Instead of choosing all 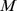 new sample points jointly, we choose 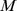 points that *independently* result in a large expected KL divergence. As a final simplification for speed and tractability, we optimize not over the entire parameter space (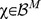), but rather select from a finite number 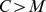 of *candidate sample points*, 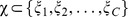, 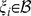. With these simplifications, the igBDOE chooses 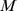 of 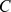 candidate points so as to maximize the sum of the expected (over outcomes) mean (over test points) KL divergence, 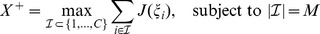
(14)where



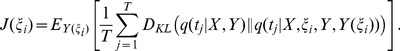
(15)Selection of the 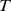 test points and 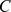 candidate points can have a significant impact on the performance of the algorithm. A simple approach is to choose these points using a LHS design. However, the resolution of test and sample points quickly becomes the limiting step as the separatrix becomes increasingly refined. To increase the density of test and sample points in places where the KL divergence is high, we propose sampling test and candidate points from the variance of the interest distribution, 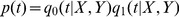
(16)

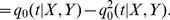
(17)


In practice, these samples are obtained using Markov chain Monte Carlo (MCMC). An optional post-processing step slightly diffuses the points by adding a random value sampled from a narrow multivariate normal distribution, having diagonal standard deviation denoted by 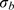, to each point from MCMC. The number of candidate points can also be varied, and in practice we find choosing 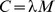 with 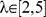 works well for all applications. Finally, to prevent the algorithm from being overly greedy when the number of observed samples is small, we initially limit the number of subsequent sample points selected by igBDOE to a fraction, 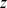, of the total number of samples, e.g. 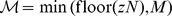. The remaining 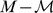 samples, if any, are obtained using LHS.

## Results

To illustrate the main contributions of this work, we have prepared a variety of results that demonstrate the Separatrix Algorithm, which has been implemented in Matlab [55]. A basic implementation of the algorithm is available in Source Code S1. We begin with a simple one-dimensional problem to show the full distribution of the estimated success probability function, and also to clearly illustrate the experiment design process for generating subsequent sample points. The methods are then applied to a two-dimensional problem with a known test function that resembles those encountered in epidemiological decision making. Finally, an agent-based stochastic simulation model of malaria [56], [57] is examined to show how the Separatrix Algorithm applies to a computer simulation having three separatrix dimensions. This simulation model was coded in C++ by a team of researchers and developers at the Institute for Disease Modeling in Bellevue, WA. Simulations were invoked on a supercomputer running Windows^®^ HPC Server with more than 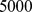 cores, however we choose 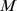 between 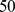 and 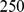 cores for these examples. The job submission pipeline and commissioning scripts use a mixture of Matlab, Python, and C#.

### Example One: Hyperbolic Tangent in 1D

The basic concepts of the method can be seen most vividly on a simple one-dimensional toy problem. Denoting by 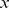 the independent parameter, the true probability of success for this example is a hyperbolic tangent, 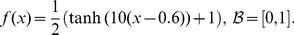
(18)


**Figure 2:**
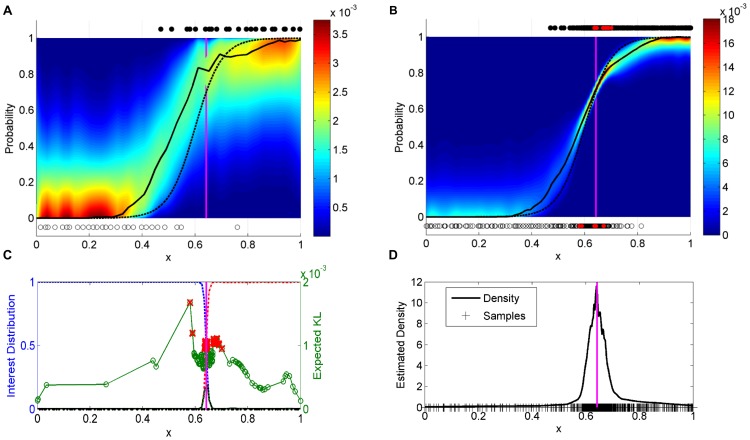
One-dimensional hyperbolic tangent analysis. (A) Shown are the true success probability function (dashed line), 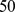 LHS samples (full and empty circles), the inferred distribution (hypercolor), and the most likely value (black line). The vertical magenta line is at the separatrix corresponding to an interest level of 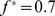. (B) The probability density after observing 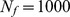 samples using the Separatrix Algorithm. Note that the estimate is tight near the separatrix. (C) The inner workings of the igBDOE algorithm. First, test and sample points are loaded from the previous iteration in which they were sampled from the variance of the interest distribution, solid black (left axis), which in turn is computed from the interest distribution: 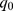 is in blue-dash and 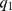 is in red dash-dot. The expected KL divergence is plotted for each of the candidate sample points (green circles, right axis). The best 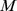 of these candidates, indicated by red crosses, will be selected. (D) The final density estimate shows that the igBDOE algorithm was placing samples in and around the separatrix. Ticks on the x-axis represent samples.

This test function is plotted in Figure 2A, along with outcomes observed at 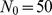 initial samples that were drawn using a LHS design. For display purposes, the posterior beta distribution (3) was evaluated at 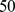 equally spaced inference points, and is displayed in hypercolor along with the mode.

For this example, we have selected a target success probability of 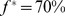, for which the sinusoidal model crosses this target at a separatrix of 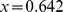. Additional samples were selected 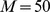 at a time until a total of 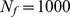 samples had been observed. At most 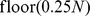 samples are added using the igBDOE algorithm per iteration, and remaining sample points come from LHS. A complete list of parameters is available in Table 1.

**Table 1:** Parameters values.

Param	Meaning	Example 1	Example 2	Example 3
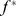	Interest level	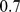	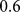	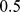
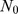	Initial number of samples	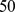	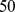	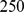
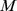	New points per iteration	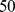	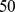	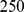
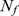	Final number of samples	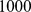	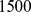	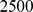
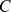	Number of candidates	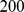	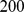	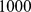
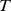	Number of test points	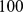	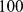	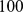
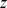	Max fraction from igBDOE	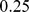	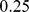	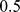
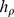	Density bandwidth scaling	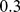	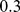	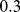
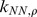	KNN for density	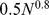	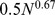	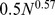
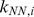	KNN for inference	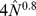	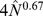	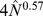
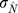	Counting kernel scale for 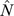	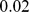	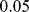	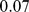
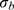	Blur kernel bandwidth	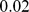	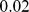	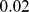

Separatrix parameter values used in the three example presented in this paper.

Upon termination, the probability density clearly reveals the location at which the test function crosses 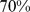, see Figure 2B. The interest-guided experiment design procedure has concentrated samples at the separatrix, see the sample point density estimate in Figure 2D.

The inner workings of the igBDOE experiment design can be seen in Figure 2C, which shows selection of the final 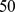 samples. Here, the interest distribution is shown as a red dashed line for 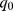 (12) and as a blue dashed-dot line for 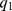 (13). The variance of the interest distribution (17), from which test and candidate points are sampled, is shown in black solid. The expected KL divergence (10) is evaluated at each candidate sample point, the green circles, and the best 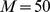 candidates, indicated by the red crosses, will be simulated on the next iteration.

To compare the performance of the Separatrix Algorithm to Latin hypercube sampling and traditional BDOE, we evaluated a metric for 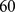 random number seeds of each algorithm. In particular, we computed the mean log likelihood at 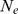 evaluation point(s), 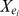, *on the separatrix*, 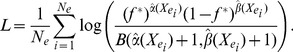
(19)


**Figure 3:**
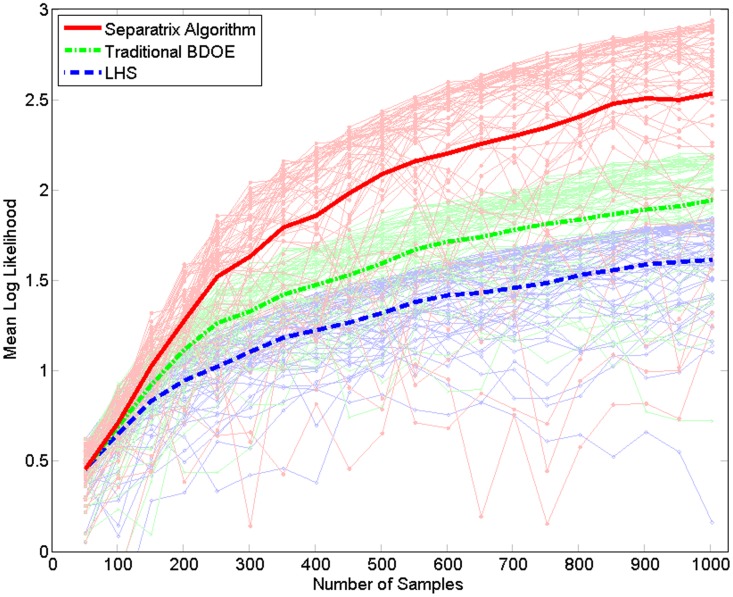
One-dimensional hyperbolic tangent performance. For the one-dimensional hyperbolic tangent test function (18), the Separatrix Algorithm outperforms Latin hypercube sampling and traditional BDOE on a likelihood-based performance metric (19).

The function denoted by 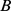 in the denominator of the log is the beta function. For this one-dimensional example, we used a single evaluation point on the 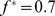 separatrix, 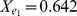. The Separatrix Algorithm outperforms LHS and traditional BDOE, as can be seen in Figure 3.

### Example Two: Hyperbolic Tangent in 2D

To extend the analysis to a two-dimensional example, we consider a test function based on a product of two hyperbolic tangents, 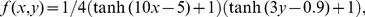
(20)where 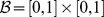.

This test function resembles certain epidemiological systems in that the probability of success is low without the interventions 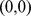 and high with both interventions at full coverage 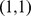 and that the probability surface can have different steepness and critical coverage for different interventions.

We configured the interest-guided experiment design procedure to select the best 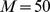 points from 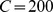 candidates, and terminated when 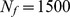. Parameters were generally similar to those used in the previous example, see Table 1.

**Figure 4:**
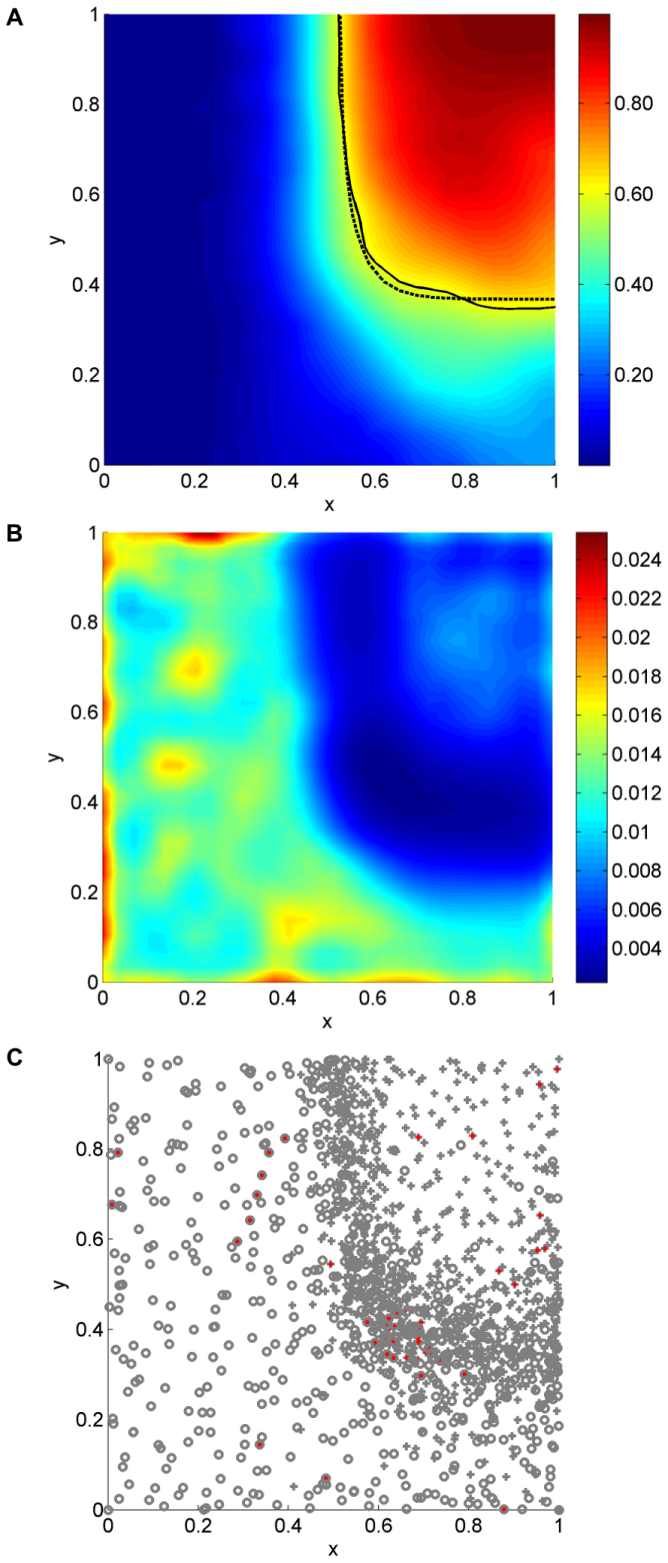
Two-dimensional separatrix results. The inference algorithm was applied at all points on a regular 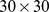 grid after collecting 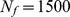 samples. Here, we display the mode (A), variance (B), and samples (C). The dashed line in (A) is the true separatrix, and the solid line is the estimate. Circles and crosses in (C) represent failures and successes, respectively, and red dots indicate samples selected on the final iteration.

The final estimate from a typical separatrix analysis is shown in Figure 4. As with the one-dimensional example, the fit is not intended to be good away from the separatrix. In fact, the interest-guided experiment design has again done well to place samples in and around the separatrix, see Figure 4C. The separatrix is more difficult to identify along the horizontal portion than the vertical portion due to the slope. The Separatrix Algorithm has correspondingly placed more samples along the horizontal portion of the separatrix, resulting in reduced variance there.

**Figure 5:**
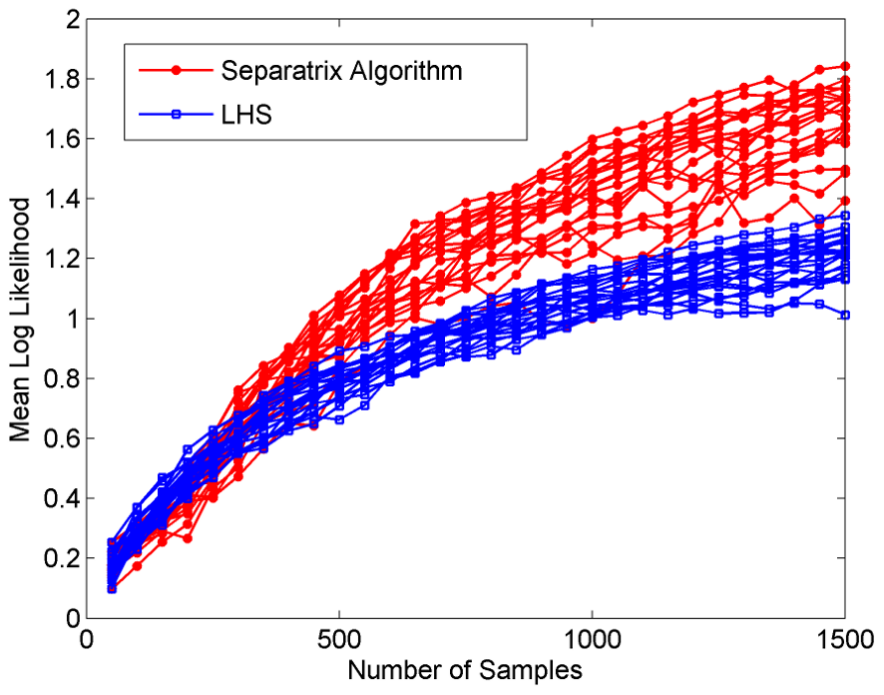
Two-dimensional hyperbolic tangent performance. The Separatrix Algorithm again outperforms Latin hypercube sampling on the mean log likelihood metric, which was evaluated at 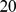 points spaced evenly in arc-length along the separatrix.

We have again compared the Separatrix Algorithm to a LHS experiment design, using the mean log-likelihood metric (19). This metric naturally balances accuracy and precision, and was evaluated at 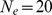 points spaced equally in arc-length along the separatrix. The results show that the Separatrix Algorithm significantly and consistently outperforms LHS, see Figure 5.

### Example Three: Individual-Based Malaria Simulation

In epidemiology, three-dimensional parameter sweeps are often conducted to explore trade-offs between competing alternatives in the context of a third parameter. We now apply the Separatrix Algorithm to a detailed computer simulation of malaria [56] for which the true response function is unknown.

This separatrix analysis considers the goal of local malaria elimination using an intervention composed of insecticide treated bednets (ITN) and a pre-erythrocytic vaccine (PEV). Each individual-based computer simulation returns true if and only if malaria is eliminated from the model within 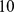 years. The success of such a campaign will depend on the local burden of disease and on the extent to which mosquitoes feed indoors. The malarial disease burden is typically quantified by the entomological inoculation rate (EIR), which quantifies the number of infectious bites received by a typical person in one year. The higher the EIR, the more difficult elimination will be. ITNs are well characterized, here we as assume that bednets are distributed to 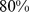 of the population, block 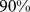 of indoor bites, and kill 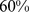 of the mosquitoes attempting indoor feeds. Naturally, bednets will not be as useful of an intervention against predominantly outdoor-feeding mosquitoes.

It is in this context that we apply the Separatrix Algorithm to explore the potential impact of a PEV, when used in tandem with ITN. Vaccines against malaria are currently under development, so it is not clear what efficacy and durability to expect. Here, we have assumed a vaccine efficacy against infection that decays exponentially with a half-life of 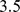 years, and use separatrix analysis to explore *initial* efficacy.

**Figure 6:**
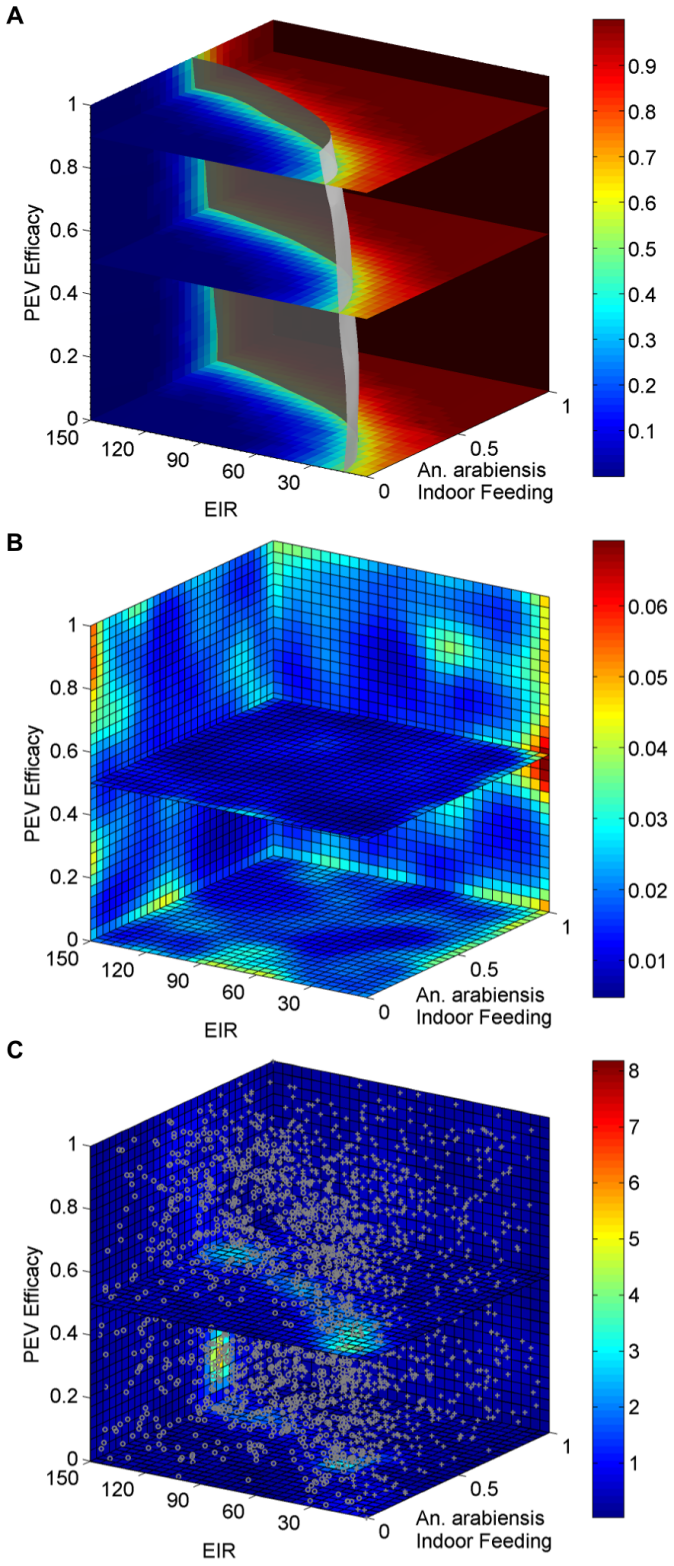
Malaria model separatrix results. The separatrix (A), variance (B), and samples with density (C) after simulating the malaria model 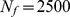 times.

We initially selected 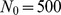 samples points using a LHS design. The Separatrix Algorithm then iteratively selected 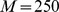 samples from 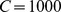 candidates. As with the previous examples, igBDOE was initially complimented by LHS. The algorithm was terminated once 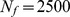 simulations had been conducted. Parameter values can be found in Table 1. The resulting separatrix plot, variance, and sample points are displayed in Figure 6.

From the separatrix plot, local elimination is possible in simulated scenario using ITN alone (PEV efficacy of zero) if the intensity of malaria transmission (EIR) is sufficiently low and mosquitoes tend to feed indoors where bed nets are effective [58]. Increasing vaccine efficacy expands the region of EIR/mosquito behavior space in which elimination is achievable.

## Discussion

To effectively leverage a stochastic simulation model in the goal-driven decision making process, we have presented the Separatrix Algorithm. The tool has potential uses in a multitude of domains, and has been discussed here in the context of infectious disease policy making. The algorithm proceeds iteratively wherein each iteration combines binary kernel regression with an interest-guided experiment design. This procedure was demonstrated on three example problems in which the Separatrix Algorithm was able to clearly isolate combinations of intervention and model parameters for which the goal is achieved with a user-specified probability of success.

In order to apply the Separatrix Algorithm, a few preliminary requirements must be satisfied. These consist primarily of a stochastic simulation with externally configurable parameters, user-specified Boolean function measuring the success of each simulation, and a target success probability isocline. Additionally, the user is required to select the region of parameter space to be explored, and it assumed that parameters not explored by the algorithm have been calibrated externally. The parameter space, 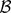, need not come directly from the input space, but instead could be mapped through a coordinate transformation to address parametric synergies. For example in malaria, immune response thresholds to within-host parasite densities and detection thresholds for within-host parasite densities can be moved together without substantially affecting the proportion detected, so some parameter explorations may choose a single dimension for both parameters. Also, the ability of the local vector population to transmit can be represented at a given point in time by a single quantity called the vectorial capacity [59].

The Separatrix Algorithm has several important limitations that should be considered. The problem being addressed by the algorithm is inherently difficult, and the complexity grows combinatorially with the number of dimensions. Therefore, separatrix exploration of a large parameter space (e.g. 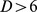, see [60]) requires access to a powerful workstation to run the algorithm and a supercomputer to run the stochastic simulations. With increasing dimensions, the number of sample points (e.g. simulations) required to achieve a specified level of confidence in the location of the separatrix increases. The computational complexity of the Separatrix Algorithm, neglecting the optional Markov chain Monte Carlo step, scales as 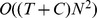. However much of the repeated computation can be cached.

The algorithm has several user-configurable parameters, including the initial number of samples, the number of samples to add on each iteration, kernel bandwidth scale factors, and so on. These parameters are inherently problem specific, and yet all three examples above were computed using similar parameters, see Table 1. Also, some analyses will be more challenging than others, depending in part on the slope of the success probability function at the separatrix. Further work is needed to determine optimal parameter values for a given problem, and automatic parameter selection could increase algorithm performance. Until an automatic procedure for selecting parameter values is available, a technician familiar with the algorithm, parameters, and implementation may be required to assist the decision maker in conducting the analysis.

More time-consuming kernel methods use complex bias reduction techniques based on pilot estimates, and cross-validation or other optimization procedures for bandwidth selection. However, the separatrix context differs from other applications because additional data can be collected. Time spent perfecting the inference or experiment design is time that could otherwise be used to generate new outcomes. For a simulation that takes days to run, it may be worthwhile to use more advanced, and computationally-intensive, kernel methods. However, for simulations taking on the order of minutes to hours, the methods we have outlined above provide a balance between experiment design and simulation.

## Conclusions

We have presented the Separatrix Algorithm for evaluating a detailed stochastic simulation with respect to a goal-oriented question and user-specified probability of success. The method combines bias-corrected kernel smoothing with an interest-guided experiment design to efficiently focus computational resources on identifying the desired separatrix. The method was applied to several toy problems representing typical response functions and to a stochastic epidemiological simulation of malaria.

Additional layers of model analysis can be included once the separatrix has been located by the Separatrix Algorithm. For example, if costing and resource allocation models are available, they can be evaluated and optimized within the separatrix. Additionally, parameters within the separatrix can be preferentially selected based on a prior or posterior parameter distribution.

The method could be improved in several ways. Numerous advances in multivariate kernel techniques have been published in the past few years. We have primarily implemented well-established techniques, but newer algorithms and automatic bandwidth selection procedures could improve the quality of separatrix plots at the cost of additional computing time. Additionally, we did not implement any correction at the edges of the parameter space. Higher-order kernels and local linear models are known to perform better at the edges.

There is no way to avoid the fact that parameter space exploration requires a considerable amount of computing power. Users lacking access to a large-scale computer should consider cloud computing, running smaller iterations, or reducing the number of dimensions. In any case, the Separatrix Algorithm uses fewer samples to achieve a desired level of precision, thereby saving time and/or money.

The experiment design procedure selects 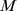 points independently. A significant performance benefit could be achieved if these points could be selected jointly, even if this means selecting only two at a time.

Finally, epidemiological models often exhibit monotonic responses. A powerful addition to the Separatrix Algorithm would be to enable user specification or automatic detection of monotonic dimensions. The approach could be based on recent advances in monotonic kernel methods [61].

## Supporting Information

Algorithm Summary S1 **Algorithm Summary S1 contains a mathematical description of the Separatrix Algorithm.**
(PDF)

Source Code S1 **Source Code S1 contains demonstration code written in Matlab.**
(ZIP)
