# Supplementary material for: The Separatrix Algorithm for Synthesis and Analysis of Stochastic Simulations with Applications in Disease Modeling
Source: PLoS One. 2014 Jul 31;9(7):e103467. doi: 10.1371/journal.pone.0103467 (PMC4117517; doi:10.1371/journal.pone.0103467)
Supplement: Algorithm Summary S1 — contains a mathematical description of the Separatrix Algorithm. (PDF) [file pone.0103467.s001.pdf]

Separatrix Algorithm Summary S1

Daniel J. Klein[1,*], Michael Baym[1,2,3], Philip Eckhoff[1]
1 Institute for Disease Modeling, Intellectual Ventures Lab, Bellevue, WA, USA
2 Department of Systems Biology, Harvard Medical School, Boston, MA, USA
3 Department of Mathematics, Massachusetts Institute of Technology, Boston, MA, USA
∗ E-mail: Corresponding dklein@intven.com

Introduction

The Separatrix Algorithm is composed of two primary sub-algorithms. The *Separatrix Inference Algorithm* algorithm estimates the distribution of the probability of success at one or more inference points. The *Separatrix Interest Guided Design of Experiments* chooses new simulation experiment to run so as to maximally gain information about the separatrix. Here, we provide a concise summary of each of these algorithmic components.

Separatrix Inference Algorithm

The inference portion of the Separatrix Algorithm can be concisely summarized as follows:

1. Compute the density of sample points at each sample point using (4).
2. Compute local sample point count at each inference point using (6).
3. Compute and apply the kernel.
   (a) The kernel bandwidth comes from (5), replacing N with $\hat{N}(x)$, the local sample point count computed above.
   (b) Compute the kernel, L_g, between sample and inference points. For all results presented in the paper, we have applied a squared exponential kernel here.
   (c) Compute $\hat{\alpha}(X^*)$ and $\hat{\beta}(X^*)$ by applying the kernel to the observed sample outcomes using (7) and (8), respectively.
   (d) Plug estimates $\hat{\alpha}(X^*)$ and $\hat{\beta}(X^*)$ into the beta distribution (3)

Separatrix Interest Guided Design of Experiments

The portion of the Separatrix Algorithm responsible for selecting M_{BDOE} new simulations to run for the next iteration, called igBDOE, can be described as follows:

1. Choose T test points and $C = \lambda M_{BDOE}$ candidate sample points:
   - For the first iteration, choose candidate and test points using LHS.
   - For subsequent iterations, load these points from the previous iteration.
2. Estimate the mode, $\bar{f}$, at each candidate sample point using the inference algorithm.
3. Compute the baseline interest distribution at all test points using (12) and (13).
4. Compute the expectation of the average (over test points) KL divergence resulting from the addition of each candidate sample point

- Loop over candidate sample points, for each candidate:
- Assume a TRUE outcome will be observed at the candidate and recompute the interest distribution at all test points. Then compute the KL divergence with respect to baseline.
- Assume a FALSE outcome will be observed at the candidate and recompute the interest distribution at all test points. The compute the KL divergence with respect to baseline.
- Compute the expectation of the KL divergence as the convex combination of the above two divergences, weighted by $\bar{f}$.

5. Select the best M_{BDOE} candidates, as ranked by the expected KL divergence.
6. Prepare and store test and candidate sample points for the next iteration:
   (a) Compute the variance of the baseline interest distribution.
   (b) Move the test and candidate points using Markov-chain Monte Carlo techniques so that the points become distributed according to the variance of the baseline interest distribution. Interpolate as needed.
   (c) If desired, apply a Gaussian blur by displacing each point by a normally distributed amount.
   (d) Save the modified test and candidate points for the next iteration.

The iteration is stopped when either the expected information gain is below a threshold or a maximum number of iterations is reached.
